# Natural Phytotherapeutic Antioxidants in the Treatment of Mercury Intoxication-A Review

**DOI:** 10.15171/apb.2018.043

**Published:** 2018-08-29

**Authors:** Velid Unsal

**Keywords:** Mercury, Antioxidants, Phytotherapy, Oxidative stress

## Abstract

Heavy metals taken into the organism can make the toxic effects on the metabolism in various ways. For example, they may interact with proteins to alter and inhibit their enzymatic and structural functions. Mercury is one of the toxic elements that are widely distributed in nature. Mercury toxicity poses a serious threat to human health. It is an element that causes oxidative stress to increase in individuals, leading to tissue damage. Oxidative stress is the result of the imbalance between the production of oxidative species and cellular antioxidant defense. Phytotherapy continues to play an important role in health care. Natural phytotherapeutic antioxidants, exhibit a broad sequence of biological impacts, including anti-oxidative stress, anti-aging, anti-toxicicity and anticancer. Many studies have also shown that the phytotherapeutic agents play an important role in the removal of mercury from the tissue and in reducing oxidative stress. Our goal in this review was to investigate alternative ways of extracting the mercury in the tissue.

## Introduction

Heavy metals do not just threaten us in our homes and in the streets. Heavy metal exposure is a serious hazard for people working in certain business lines. The definition of heavy metals is in fact used for metals with a density greater than 5 g / cm^3^. This group contains more than 60 metals including lead, cadmium, mercury, chromium, nickel iron, cobalt, copper and zinc. Free radical species are molecules that consistently occur in organisms and are regularly removed by the antioxidant defense system.^1-3^

Phytotherapy is a natural treatment method that is benefited from the plants that gain importance in health field. It has an important place in the prevention or reduction of diseases. The reason for people's trust in phytotherapy is that plants are natural, unlike synthetic drugs. Numerous studies have been conducted with natural antioxidant plants.^4-6^ Exposure to mercury in vivo and in vitro increases ROS and RNS. Increased ROS and RNS at high concentrations can damage biomolecules. In recent years, the role of natural antioxidants in reducing ROS / RNS damage and their therapeutic potential has increased the interest of people in these. In addition, the absence of antioxidant structures causes an increase in ROS / RNS concentrations.^7-10^ Our purpose in this review is to discuss the role of natural antioxidants in reducing mercury damage.

## Hg (Mercury)

Mercury (Hg) is a silver, fluid, bright, odorless heavy metal. The symbol is "Hg" and the atomic number is 80. The symbol Hg "hydrated silver" comes from the Latin term hydrargyrum. Mercury is a stable element with a valence of +2.^11,12^ Metallic or elemental mercury (Hg°) are the naturally occurring main forms of mercury. In nature, elemental mercury is found in the form of organic and inorganic compounds. The industry is mainly used in medical devices such as mercury fluorescent lamps, blood pressure monitors and thermometers used in many areas. It is widely used as filler material in dentistry, mine melting, cement making and paper production.^13,14^ In a study conducted in the United States, mercury was reported to be the third most common environmental metal.^15^ Mercury exposure can occur through respiration, feeding and food chain. Mercury is a heavy metal that is extremely toxic, which can have multiple adverse effects, and ultimately leads to cell death. Mercury, dysfunction in the skeleton of the cell and in the endoplasmic reticulum, significant cytoplasmic acidosis causes loss of mitochondrial function.^16^ Chemically, mercury and its compounds can be examined in 3 different categories, (As shown in Table 1). Elemental (metallic) Mercury; Elemental mercury may evaporate at room temperature. Elemental mercury may evaporate at room temperature. And steam is rapidly absorbed from the lungs and it spreads to the central nervous system.^17^ Inorganic mercurials include ammoniated mercury(ClH_2_HgN), mercuric chloride (HgCl_2_), mercuric oxide (HgO), mercuric sulfide, mercuric iodide (HgI_2_) , and the phenylmercuric salts. (C_8_H_8_HgO_2_) Organic mercurials include ethylmercury (C_2_H_5_Hg^+^), methylmercury (CH_3_Hg^+^), thimerosal (merthiolate), and merbromin (C_20_H_9_Br_2_HgNa_2_O_6_ -mercurochrome).^18,19^ Methyl and phenyl mercuric compounds, such as metallic mercury, have the ability to cross the blood-brain and placental barrier. The lipophilic nature of the metallic mercury allows for distribution throughout the body.^20,21^

**Table 1:** Simple general informations about mercury^22^

**Mercury**	**Resources**	**Routes of Exposure**	**Excretion and excretion**	**Toxicity**
**Elemental Mercury**	Gold mining, Dental amalgam, Thermometers, and other measuring instruments, volcanoes, Combustion	Inhalation	Urine and stool	Nervous system Kidney, Lungs, Skin
**Inorganic Mercury**	Thiomersal, Cosmetics, Lambs, Photography, disinfectants	Digestion, Dermal	Urine	Nervous system, Kidney, Digestive system, Skin
**Organic Mercury**	Fishes, Fungicide	Digestion, parenteral, transplacental	Stool	Nervous system, Cardiovascular

## Mercury toxicity manifests itself in various mechanisms

**Respiratory system findings:** In humans, acute and high levels of metallic mercury vapour have significant effects on the respiratory system. The most reported effects are cough, constriction with dyspnea, or burning sensation.^23,24^

**Cardiovascular findings:** Exposure to all conditions increases the heart rate and blood pressure.^25,26^

**Gastrointestinal findings:** The most obvious sign of mercury intoxication is the burning sensation of oral mucosa. Other gastrointestinal effects at high levels of acute exposures are abdominal pain and diarrhea.^23,24,27,28^

**Renal findings:** A sensitive target organ is the kidney in exposure to metallic mercury inhalation. Mercury has been found to cause nephrotic syndrome or tubular damage by tubular dysfunction. The cause of nephrotic syndrome due to mercury is an autoimmune reaction.^23,24,29,30^

**Neurological system findings:** In humans, adverse neurological effects have been reported following high concentration acute mercury vapor inhalation. Generally, perceptual, personal, conceptual, and motor confusion are reported. The most important symptoms are tremor, emotional tenderness, insomnia, memory loss, neuromuscular changes (weakness, muscle atrophy and muscle withdrawal), headache and polyneuropathy.^23,31-35^ Exposure to mercury compounds at an early stage causes long-lasting and permanent neurobehavioral and neurochemical irregularities. Like Parkinson's disease and Alzheimer's disease.^36^

The elimination of mercury from the body is very difficult. Following metallic mercury exposure, the elimination urine, feces and respiration.^37-38^ Experimental studies with neural cells in vitro have demonstrated that mercury induces glial cell reactivity (a distinctive feature of brain inflammation) and increases the expression of amyloid precursor protein.^39^

**Endocrine findings:** Thyroid function tests (TFTs) are thought to change levels. (Free-T3 and Free-T4). The most affected hormones by mercury are insulin, estrogen, testosterone and adrenaline.^40-43^

**Dermatological findings:** Acute and moderate exposure to elemental mercury vapor via inhalation results in erythematosus and pruritic skin diseases.^18,44,45^ In people with tattoos containing red pigment of the origin of mercuric sulfur (cinnabar-vermilion, Chinese red), they may experience inflammation that is limited to this region within 6 months of tattooing.^18,46^

**Inflammation findings:** Inflammation caused by the influence of heavy inorganic mercury causes the tendency to bleed in gingiva and oral mucosa. It increases the salivary secretion, causing sensation of metallic taste in the mouth. Gingiva, a gray line is formed, especially when the oral hygiene is bad.^29^

**Birth defects and reproductive system:** There is also evidence that mercury poisoning leads to Young syndrome (bronchiectasis, low sperm count, impairs sperm quality.).^47-49^

It has been determined that all forms of the mercury can pass to the placenta at varying gauges^50^ Mercury exposure is dangerous for the baby, because the baby's neurological tissues develop during early gestation.^51^

**Immune system findings:** Mercury compounds reduce the number of T lymphocytes.

In addition, mercury exposure causes a decrease in T cell GSH content. Mercury is an immunotoxic agent.^52-54^

In the case of inorganic mercury exposure, elimination occurs via urine and faeces. Organic mercury compounds predominantly excrete in humans.^11^ Mercury, promotes the formation of reactive oxygen species (ROS) such as hydrogen peroxides.^55,56^

## ROS (Reactive Oxygen species)

Free radicals are highly active atoms or molecules that can be produced in many physiological and pathological processes, carrying one or more unpaired electrons in their orbit. These highly unstable atoms or molecules tend to react with molecules in their environment and to share these electrons.^57,58^ Free radicals can be positively charged, negatively charged, or neutral and are most often formed by electron transfer in biological systems. The most important free radicals in biological systems are the oxygen radicals. In addition, another source of free radicals is the nitrogen molecule. There is no toxic effect of O_2_, but it becomes free oxygen radicals during aerobic cell metabolism. By partial reduction of O_2_, OH and O_2_ -are formed.^58-60^

## Superoxide Radical (O2^•–^)

Superoxide is the first radical to appear in living organisms. In almost all aerobic cells, reduction of oxygen by an electron takes place.^61^ The superoxide radical plays an important role in the formation of other reactive oxygen species, such as H_2_O_2_, HO_2_^-^ or ^1^O_2_.^58,62^

The superoxide radical is produced either directly in mitochondria during oxidation or enzymatically by xanthine oxidase (XO), cytochrome p450 and other oxidases. Superoxide dismutase (SOD) enzyme or in H_2_O_2_ is spontaneously inactivated.^63,64^ Combined with superoxide (O2^•–^) and the free radical NO-, comes the reactive nitrogen derivative ONOO^.^(Peroxynitrite). ONOO- has harmful effects on direct proteins.^65^

## Hydroxyl radical (•OH)

The hydroxyl radical is the most reactive radical. It reacts with lipids, polypeptides, proteins, DNA and other molecules (such as thiamine and guanosine).^62,66,67^

## Singlet oxygen (^1^O_2_)

Singlet oxygen is a nonradical and induced status. Compared with other ROS, singlet oxygen is rather mild and non toxic for mammalian tissue.^68^ ^1^O_2_ is a cell signal and messenger; redox active agents regulate ion channel activity in animals and plants. In the human organism, singlet oxygen is both a signal and a weapon, with therapeutic potency against various pathogens such as microbes, viruses, and cancer cells.^62^

## Hydrogen peroxide (H_2_O_2_)

There is no unpaired electron in the hydrogen peroxide molecule, and so it is not a radical. Hydrogen peroxide can be generated through a dismutation reaction from superoxide anion by superoxide dismutase (SOD).^58,62,63^

Enzymes such as amino acid oxidase (AAO) and xanthine oxidase (XO) also produce hydrogen peroxide from the superoxide anion. H_2_O_2_ is the least reactive molecule among ROS and is stable under physiological pH and temperature in the lack of metal ions. H_2_O_2_ can produce singlet oxygen thanks to react with superoxide anion or with HOCl or chloramines in living systems.^62,68^ Free radical species (ROS) affect all important structures of cells such as proteins, carbohydrates, lipids, DNA and enzymes.^58,69^

## Effects of ROS on lipids and proteins

Free radicals have to cross the cell membrane in order to interact with the cell components. Inasmuch as cell membranes are rich in polyunsaturated fatty acids (PUFAs) and cholesterol, they are easily affected by oxidant radicals.^70^ Free radicals move away the hydrogen atom from the fatty acid chain. Lipid peroxidation is oxidation of polyunsaturated fatty plural form with free radicals. The main primary products of lipid peroxidation are lipid hydroperoxides (LOOH). This peroxidation results in products such as MDA, 4-hydroxynoneal (HNA), 8-iso-prostaglandin F_2α_ (8-iso-PGF_2α),_ alcohols, ethane and pentane. MDA is mutagenic since DNA can react with nitrogen bases.^58,71^ Additionally it is genotoxic and carcinogenic on cell cultures. Membrane damage caused by lipid peroxidation is irreversible. MDA is the most mutagenic product of lipid peroxidation. 4-HNE is the most toxic. 4-HNE is considered as the second toxic messengers of free radicals, one of the major generators of oxidative stress and a major lipid peroxidation product.^58,70,72^ 8-Iso-prostaglandin F2α (8-iso-PGF2α), a major F2-isoprostane, is biosynthesized in vivo through nonenzymatic free radical-catalysed peroxidation of arachidonic acid.^21,22,58,70,73,74^

Proteins are defined as the major targets of oxidative damage. The products of cellular metabolism or environmental induced ROS changes in the amino acids of proteins and cause loss of activity of protein function / enzymatic activity as well.^75,76^

Oxidative protein modifications may take place in different ways. ROS directly interacts with protein or Interaction of compounds such as carbohydrates, lipids, and nucleic acids with ROS can interact with proteins with the resulting products.^77^

In this way, reactive and non-protein compounds react with proteins to form a wide array of structures.^78,79^ Protein oxidation reactions are usually divided into modifying the protein construct and modifying the amino acid side chains.^80,81^ In addition to the modification of the protein in proteins, amino acid side chains are target for ROS. Sulfide containing amino acids in the structure are highly sensitive to cysteine and methionine.^82^ Aromatic structures are also the main targets for ROS. The oxidatively modified tyrosine, phenylalanine and tryptophan are usually oxidative damage a demonstration.^76^ Oxidation of lysine, arginine, proline or threonine may result in the formation of carbonyl derivatives.^83,84^ Protein carbonyls are among the most commonly used products for determination of the proteins of the oxides.^58,85^ Protein carbonyl levels area well-used marker for oxidative stress. The toxic effects of mercury can be prevented by antioxidant defense mechanisms to a certain extent.

## Reactive nitrogen species (RNS)

NO reacts with the superoxide radical or molecular oxygen, leading to the formation of various reactive intermediates called reactive RNS. RNS consist of nitrite derivatives such as NO, NO_2_ - and OONO -.^86^

## Nitric oxide (NO•)

Nitric Oxide (NO) is highly stable at high concentrations in an oxygen-free environment and stable at low concentrations in the presence of oxygen. NO is a signal molecule of low molecular weight known to be biologically active in mammalian cells. The NO radical is synthesized from L-arginine by nitric oxide synthase (NOS) enzyme catalysis in vascular endothelial cells.^87^ NO is an important effector and messenger molecule that plays a role in various biological processes such as immune response, smooth muscle tone, apoptosis, angiogenesis and nervous system.^88^ In addition, NO is a molecule that regulates numerous pathological and physiological states.^89^ NO has very important physiological functions at low concentrations. NO binds with molecular oxygen to form nitrogen dioxide (NO2). Another important effect of NO is to produce a strong oxidant peroxynitrite (ONOO -^.^).^90^ NO is a mediator with autocrine and paracrine effects in hemostatic events and in the defense mechanisms of the organism. The most important function of NO is to function in parallel with the effects of interleukin-1 (IL-1) and cytokines in various tissues of the body. It is produced by macrophages, neutrophils, hepatocytes and endothelial cells.^91^ However, at high concentrations it shows toxic effects on normal cells. Spontaneously decomposes to form nitrogen dioxide.^92^ In mammals, NO can be produced with three different isoforms of enzymes NO synthase. NOS enzymes are found in two basic isoform. These; is called constitutive or structural (cNOS) and inducible (iNOS). Structural NOS enzymes have two isoforms, endothelial NOS (eNOS) and neuronal NOS (nOS). eNOS is located on the membrane and is synthesizing the endothelium-induced relaxation factor; nNOS produces NO2, the messenger molecule in the central nervous system and neurons. Structural NOS is Ca ^+2^/ calmodulin dependent as cofactor and produces small amounts of NO at intervals with low activity.^93,94^

## Peroxynitrite (OONO-)

Peroxynitrite is an important biological oxidant formed by the reaction of nitric oxide and superoxide radicals**.** Peroxynitrite can cause oxidative damage, nitration, and S-nitrosylation of biomolecules including proteins, lipids, and DNA.^93^ Peroxidation of lipids in the membrane distorts membrane integrity by reducing the fluidity, elasticity and permeability of the cell membrane. These radicals constantly increase the level of Ca^2+^ in the cell and cause cytotoxic effect on the cell by inhibition of mitochondrial respiration and electron transport chain, decrease of ATP production and activation of radical generating enzymes.^95,96^

## Antioxidants

Although free radical reactions are necessary for the defense mechanism of neutrophil, macrophage and other immune system cells, they result in overproduction of free radicals, tissue damage and cell death. The half life of ROS is short. However, they initiate free radical chain reactions that cause tissue damage. For this reason, defensive mechanisms against oxidative damage triggered by free radicals act. These are preventive mechanisms, repair mechanisms, physical defenses and antioxidant defenses.^57^ Antioxidant defense is the prevention or delay of oxidation of oxidizing agents such as proteins, lipids, carbohydrates and DNA in living cells. The substances that play a role in this process are called 'antioxidants'.^97,98^ Enzymatic antioxidants are Superoxide dismutase (SOD), Catalase (CAT), Glutathione peroxidase (GSH-Px), Glutathione reductase (GR), Glutathione S-Transferase (GST) ,enzymes. The SOD structure contains copper (Cu), zinc (Zn) and manganese (Mn). GSH-Px contains selenium ions. For this reason, these enzymes are called metalloenzymes.^99,100^ In contrast to the intracellular environment, E and C vitamins, transferrin, ceruloplazmin, albumin, bilurubin, β-carotene are responsible for the non-enzymatic antioxidant defense in the extracellular environment. In addition, E and C vitamins have antioxidant properties within the cell.^58,65,72,101^

## Enzymatic antioxidants

**Superoxide dismutase (SOD):** By catalyzing the conversion of O_2_•-radical to H_2_O_2_, protects the cells from harmful effects of O_2_•-radical. It also inhibits lipid peroxidation. SOD plays a role in controlling the levels of **O2**^•–^in the parts of the cell Superoxide dismutase activity varies with tissues. It is mainly intracellular and 10% is located outside the cell.^58,102,103^

**a**- **Copper and zinc containing (Cu-Zn SOD) dismutases (cytosolic SOD):** It contains copper and zinc in its active site. The enzyme is located in the cytoplasm of the cells. Cyanide is an inhibitor of this enzyme.^104,105^

**b**- **Manganese superoxide dismutase (Mn SOD):** Mitochondrial Mn-SOD is a homotetramer containing one Manganese atom per subunit. Mn-SOD has the same reaction as Cu-Zn-SOD. However, it is a completely different enzyme in its structure. It contains Mn in its active site and is not stable. This form of SOD is not inhibited by cyanide.^105^

**Catalase (CAT):** Catalase is a hemoprotein that has four groups in its structure. Catalase converts hydrogen peroxide (H_2_0_2_) to water (H_2_0). Catalase's effect is similar to SOD.^106^

H_2_O_2_ + H_2_O_2_→H_2_O+1/2O_2_

**Glutathione peroxidase (GSH-Px):** GSH-Px can be found in two forms, selenium-bound and selenium-bound. Selenium based group, reducing hydrogen peroxide and other organic peroxides. It consists of four members. These are GSH-Px1 (celluler- GSH-Px), GSH-Px2 (GSH-Px-gastrointestinal), GSH-Px3 (plasma-GSH-Px) and GSH Px4 (PH-GSH-Px), respectively.^107^

- GSH-Px1 or cellular GSH-Px (cGSH-Px), tetrameric in structure is a cytosolic enzyme. GSH-Px1 is active against organic hydroperoxides and H_2_O_2_.^108^
- GSH-Px 2, or gastrointestinal GSH-Px (GSH-Px-GI) found in the gastrointestinal tract, but not in the kidney, heart and lung.^109^
- GSH-Px 3, A glycoprotein isolated from the lipid portion of the plasma. It is found in the lung, plasma and other extracellular fluids.^110^
- GSH-Px 4 is expressed in the liver and gastrointestinal tract in humans. GSH-Px 4 or phospholipid GSH-Px (PH-GSH-Px) is found in cytosol, mitochondria and cell membrane. The enzyme reduces the phospholipid hydroperoxides to alcohols and protects the membrane against peroxidation in the absence of the most important antioxidant E vitamin.^111^

**Glutatyon S-transferaz (GST):** Glutatyon S-transferazlar (GSTs) catalyze the nucleophilic attack of glutathione (GSH) tripeptide on electrophilic substrates in catalysis reactions.^112^ Phase-II detoxification is a member of the enzyme family. In addition, it prevents oxidative products or foreign toxic substances from merging with other macromolecules in the body and provides them to be removed without harming the cell components. Therefore, GSTs are one of a group of enzymes that are very important guardians.^113^ GSTs are divided into three families as mitochondrial, cytosolic and microsomal.^114^ Mercapturic acid plays an important role in the initial reactions of biosynthesis. The mercapturic acid formation process catalyzed by GSH-conjugation of GST is generally described as detoxification reactions. The ability to reduction feature GSTs protects membrane components from lipid peroxidation. In addition, 4-hydroxy alkenals, products of lipid peroxidation in aldehyde structure, are conjugated with GSH.^115^ GSTs, also considered as one of the natural protective systems, have an important role in the detoxification of electrophilic xenobiotics such as herbicides, pesticides, anticancer drugs, chemical carcinogens and environmental pollutants.^116^

**Glutathione reductase (GR):** Glutathione reductase is an antioxidant enzyme that converts oxidized glutathione (GSSG) to reduced glutathione (GSH). GR uses NADPH as the coenzyme when performing catalysis.^117^ The physiological GSH-GSSG ratio in the cells is of great importance. In the absence of GCSG, the level of intracellular NADPH is reduce and GR is inactivate. As the intracellular level of GSSG increases due to the oxidative stress, GR re-activates.^118^

## Nonenzymatic antioxidants

Nonenzymatic antioxidants; It is examined in two parts as natural antioxidants and synthetic antioxidants. This review will discuss natural antioxidants. For this reason, synthetic antioxidants were excluded from the discussion.^119^

**Glutathione (GSH):** GSH is made from three amino acids: glycine, cysteine and glutamic acid. This tripeptide exists in reduced (GSH) and oxidized (GSSG) forms. The relative amounts of every form determine the cellular redox status (GSH/GSSG ratio) which is often used as a sign of antioxidative capacity of cells. Glutathione (GSH) has vital importance in fighting oxidative stress. It is a strong free radical and reactive oxygen species scavenger.^120,121^

**Vitamin E:** Vitamin E has eight isoforms, α-, β-, γ-, and δ-tocopherol and α-, β-, γ-, and δ-tocotrienol. Vitamin E is a lipophilic radical-scavenging antioxidant.^122^

**Vitamin C:** Vitamin C is a potent antioxidant protecting the body against endogenous and exogenous oxidative challenges.^123^

**Uric acid:** Uric acid demonstrated its ability to scavenge reactive radicals resulting from harmful process, such as autoxidation of hemoglobin, or peroxide generation by macrophages. it is an efficient scavenger of singlet oxygen, peroxyl and hydroxyl radicals and protects erythrocyte membrane from lipid peroxidation.^124^

**Retinoids and carotenoids:** Retinoids and carotenoids take place in the structure of lipids and cell membranes. In the singlet oxygen suppression to prevent the harmful effects of flavin and porphyrin, they work in preventing peroxide radicals.^101^

## Discussion

Plants are an exogenous source of antioxidants taken in the diet. It is believed that two thirds of the plant species in the world have medical prescription, and almost all of them have excellent antioxidant potential.^125^ Increased exogenous antioxidant supplementation or endogenous antioxidant defense has been found to be effective in combating undesirable effects of oxidative stress.^126^ The main natural antioxidants present in vitamins and protecting the human body from harmful free radicals are mainly vitamins (C, E and A vitamins), flavonoids, carotenoids and polyphenols.^127^ Phenolic compounds exhibit physiological properties such as anti-allergic, anti-atherogenic, antimicrobial, anti-inflammatory, antioxidant, anticancer, antithrombotic, cardiovascular and vasodilatory effects.^128-130^

In addition, Fruits, spices and many medicinal herbs are rich sources of pharmacological properties. These agents have antioxidants, free radical scavengers and anti-toxic properties.^16,23,131-132^

As shown in Table 2, many natural antioxidants against mercury poisoning have been tested for detoxification.

**Table 2:** Some antioxidants used for treatment of mercury poisoning

**Models**	**Study Design**	**Materials**	**Effect**	**Mechanisms and Conclusion**	**References**
HgCl_2_ ; (12 µmol kg^−1^ b.w.)	Wistar albino rats	Curcumin- 80 mg kg^−1^ b.w , orally	MDA ↓, GSH-Px ↑ CAT ↑	Oxidative stress inhibitor.	^133^
HgCl_2_ ; (1 mg/kg bw)	Wistar albino rats	Sodium selenite (0.25 mg/kg bw ) and/or vitamin E (100 mg/kg bw) +	CAT ↑,GSH-Px↑,SOD↑, MDA↓	Sodium selenite and/or vitamin E could ameliorate the mercury induced testicular toxicity.	^134^
MeHgCl; (5 mg/kg bw/day)	Male albino rats	α-linolenic and α-eleostearic acid	SOD ↑ CAT↑ ,GSH ↑, MDA ↓	Both are protective against mercury toxicity.	^135^
HgCl_2_ (80 mg/L)	Wistar albino rats	Luteolin	MDA ↓; GSH ↑,GSH/GSGG↑	Luteolin eliminates ROS and prevents the induction of HgCl_2_ in antioxidant defenses.	^136^
HgCl_2_ (12 μmol/kg)	Sprague-dawley rats	*Zingiber officinale* (125 mg/kg) and 6-gingerol (50 mg/kg)	GSH-Px ↑ CAT ↑,GSH-Px↑,SOD↑, MDA↓,GST↑	Both are protective against inorganic mercury toxicity.	^137^
HgCl_2_ (5 mg kg^-1^b.w)	Wistar albino rats	*Moringa oleifera oil* (2 ml kg^−1^b.w) and (Coconut oil 2 ml kg^−1^ b.w)	GSH-Px ↑ CAT ↑,GSH ↑,SOD↓, MDA↓,	Moringa oleifera oil and Coconut oil was ameliorated the HgCl_2_ induced testicular toxicity.	^138^
HgCl_2_; (0,4 mg/kg b.w)	Wistar albino rats	Berberine (100 mg/kg b.w)	SOD↑, CAT↑ , GSH ↑ ; MDA ↓ GR ↑	Berberine reduced HgCl_2_-induced neurotoxicity. Berberine has a therapeutic potential as a neuroprotective agent.	^139^
HgCl_2_; (2 mg/kg)	Male sprague-dawley rats	Rhubarb (1200 mg/kg), Anthraquinones (200 mg/kg), Total tannins (TT, 780 mg/kg)	GSH-Px ↑	Rhubarb play a protective role against HgCl_2_-induced acute renal failure. Rhubarb can be developed as an antidote.	^140^
HgCl_2_; (50 g/kg/b.w)	Wistar strain albino rats	Diallylsulphide (DAS) 200 mg/kg/b.w	SOD↑, CAT↑ , GSH-Px ↑	DAS shows antioxidant activity and plays a protective role against mercury-induced oxidative damage in the rat livers.	^141^
HgCl_2_; (5 mg/kg)	Wistar albino rats	*Ginkgo biloba* extract 150 mg/kg daily i.p. for 5 days	MDA ↓, GSH ↑	Oxidative damage of HgCl_2_ in brain, lung, liver and kidney tissues is protected by antioxidant *Ginkgo biloba* extract.	^142^
HgCl_2_; (5 mg/kg i.p)	Wistar albino rats	*Aralia elata* polysaccharide (100 mg/kg daily i.p)	CAT↑,SOD↓,MDA↓,GST↑,MPO↑	*Aralia elata* polysaccharide may afford the protection against HgCl_2_-induced cardiovascular oxidative injury in rats.	^143^
HgCl_2_; (4.0 mg/kg)	Male wistar rats	*Eruca sativa* 50, 100 and 200 mg/kg/b.w	GSH-Px ↑ CAT ↑,GSH ↑,SOD↓, GR ↑,MDA (TBARS)↓	E. sativa seeds to possess a potent antioxidant and renal protective activity and preclude oxidative damage inflicted to the kidney.	^144^
HgCl_2_ (0.8 g/kg)	Male wistar rats	*Urtica dioica* 1.5 ml UD/rat	GSH ↑	Fresh nettle leaves are a protective plant that can play a beneficial role in preventing Hg poisoning.	^145^
HgCl_2_; (5 mg/kg b.w)	Mice	*Piper cubeba* 200mg/kg b.w	CAT↑,,MDA (LPO)↓,GST↑, GSH-Px ↑	*Piper Cubeba* extract improves antioxidant status by increasing GSH-Px, CAT activity and GSH levels in the liver.	^146^
HgCl_2_; 2 mg/kg orally single dose	Mice	*Tribulus terrestris extract* 5.0 mg/kg	MDA (LPO- lipid peroxidation)↓, GST↑,	Tribulus terrestris, may afford the protection against acute HgCl_2_ toxicity, by reduction of free radical accumulation and GSH depletion.	^147^
HgCl_2_; (1.29 mg/kg b.w)	Male wistar rats	Gallic acid 30 mg/kg b.w	CAT↑,MDA (LPO)↓,GST↑, GSH-Px ↑, SOD↑,	Gallic acid may increase antioxidant activities and nullify the toxicity effect of mercury toxicants.	^148^
HgCl_2_(80 mg/L with water)	Male wistar rats	Luteolin (80 mg/kg per day in 1% dimethylsulfoxide (DMSO) intragastrically)	MDA↓,GST↑	Luteolin supplementation reduces renal mercury accumulation. Therefore, luteolin can serve as an alternative treatment to prevent renal damage.	^149^

## Conclusion

In summary, this study provides evidence that some natural antioxidants play a protective role against Mercury-derived toxicity. It provides foundation of studies of natural phytotherapeutic agents on mercury treatment. This study also provided information for candidate antidote, pharmaceutical agents in the treatment of mercury-induced toxicity. However, high doses of antioxidant supplements often do not work well or can be harmful. More research is needed for effective and safe antioxidant doses against mercury poisoning.

## Ethical Issues

Not applicable.

## Conflict of Interest

The authors declare no conflict of interests.
